# Violet to Near‐Infrared Optical Addressing of Spin Pairs in Hexagonal Boron Nitride

**DOI:** 10.1002/adma.202414846

**Published:** 2025-02-18

**Authors:** Priya Singh, Islay O. Robertson, Sam C. Scholten, Alexander J. Healey, Hiroshi Abe, Takeshi Ohshima, Hark Hoe Tan, Mehran Kianinia, Igor Aharonovich, David A. Broadway, Philipp Reineck, Jean‐Philippe Tetienne

**Affiliations:** ^1^ School of Science RMIT University Melbourne VIC 3001 Australia; ^2^ Takasaki Institute for Advanced Quantum Science National Institutes for Quantum Science and Technology Takasaki Gunma 370‐1292 Japan; ^3^ Department of Materials Science Tohoku University 6‐6‐02 Aramaki‐Aza, Aoba‐ku Sendai 980‐8579 Japan; ^4^ ARC Centre of Excellence for Transformative Meta‐Optical Systems, Department of Electronic Materials Engineering, Research School of Physics The Australian National University Canberra ACT 2600 Australia; ^5^ School of Mathematical and Physical Sciences University of Technology Sydney Ultimo NSW 2007 Australia; ^6^ ARC Centre of Excellence for Transformative Meta‐Optical Systems University of Technology Sydney Ultimo NSW 2007 Australia; ^7^ ARC Centre of Excellence for Nanoscale BioPhotonics, School of Science RMIT University Melbourne VIC 3001 Australia

**Keywords:** hexagonal boron nitride, optical emitter, spin defect, optically detected magnetic resonance

## Abstract

Optically addressable solid‐state spins are an important platform for practical quantum technologies. Van der Waals material hexagonal boron nitride (hBN) is a promising host as it contains a wide variety of optical emitters, but thus far observations of addressable spins have been sparse, and most of them lacked a demonstration of coherent spin control. Here, robust optical readout of spin pairs in hBN is demonstrated with emission wavelengths spanning from violet to the near‐infrared. It is found that these broadband spin pairs exist naturally in a variety of hBN samples from bulk crystals to powders to epitaxial films, and can be coherently controlled across the entire wavelength range. Furthermore, the optimal wavelengths are identified for independent readout of spin pairs and boron vacancy spin defects co‐existing in the same sample. These results establish the ubiquity of the optically addressable spin pair system in hBN across a broad parameter space, making it a versatile playground for spin‐based quantum technologies.

## Graphical abstract

Spin‐dependent optical emission is observed in hexagonal boron nitride across a wide band of wavelengths from the violet to the near‐infrared, and attributed to spin defect pairs. These broadband spin pairs are found to exist naturally in a variety of samples from bulk crystals to powders to epitaxial films, and can be coherently controlled across the entire wavelength range.

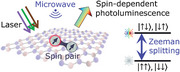

## 1 Introduction

Van der Waals materials offer unique opportunities for quantum technologies due to the ability to exfoliate and integrate them into complex multi‐functional heterostructures and devices.^[^ ^1^ ^]^ In particular, hexagonal boron nitride (hBN) has emerged as a front runner for photonic and spin‐based quantum technologies.^[^ ^2^, ^3^ ^]^ With its wide bandgap (≈6 eV), hBN hosts a rich library of optically active defects,^[^ ^4^, ^5^, ^6^, ^7^ ^]^ some of which have been observed at the single defect level thereby serving as bright single‐photon emitters.^[^ ^8^ ^]^ These optical emitters span a broad range of emission wavelengths, including UV emitters (≈300 nm,^[^ ^9^, ^10^, ^11^ ^]^) violet‐blue emitters (≈430 nm,^[^ ^12^, ^13^, ^14^ ^]^) visible emitters from green to red,^[^ ^15^, ^16^, ^17^, ^18^, ^19^, ^20^, ^21^ ^]^ the boron vacancy ($V_{B}^{-}$) defect which emits around 800 nm,^[^ ^22^ ^]^ and other near‐infrared (NIR) emitters up to ≈1000 nm.^[^ ^23^ ^]^ Among these colour centres, the $V_{B}^{-}$ defect is the only one that had its atomic structure unambiguously identified. Moreover, $V_{B}^{-}$ has a paramagnetic spin‐triplet ground state which can be initialised and read out optically, a property that has attracted significant interest for quantum technologies.^[^ ^24^, ^25^, ^26^, ^27^, ^28^, ^29^, ^30^ ^]^ However, the $V_{B}^{-}$ defect is very dim and as a result it has only been observed in ensembles thus far. The other emitters are typically much brighter than $V_{B}^{-}$ and a subset are believed to be associated with carbon impurities,^[^ ^31^ ^]^ though their exact atomic structures remain unknown. Interestingly, some of the visible emitters have been reported to possess an addressable spin doublet and are measurable as single isolated emitters,^[^ ^31^, ^32^, ^33^, ^34^, ^35^, ^36^, ^37^, ^38^, ^39^ ^]^ making them extremely promising as qubits for quantum technologies. As we showed in ref. [38], these optically addressable spin systems correspond to pairs of weakly coupled electronic spins carried by two nearby point defects, one of which is optically active. However, most studies to date have focused on the yellow‐orange emission region (≈600–700 nm,^[^ ^31^, ^33^, ^35^, ^36^, ^37^, ^38^, ^39^ ^]^) with just one report of green emitters in a nanopowder sample (540 nm^[^ ^34^ ^]^) and of red emitters in an exfoliated flake (720–760 nm,^[^ ^32^ ^]^) and most of these reports have not shown coherent spin control. Whether this family of spin‐active emitters can be reproducibly observed and coherently controlled across many different hBN samples, let alone extended toward the UV and NIR ends of the spectrum, remains unknown. Additionally, although spin‐active visible emitters have been shown to co‐exist with $V_{B}^{-}$ defects^[^ ^36^ ^]^ when excited by the same laser, independent optical readout of two distinct spin species would open intriguing possibilities for quantum technologies.

In this paper, we show that optically addressable spin pairs exist in hBN with emission wavelengths continuously ranging from the violet (420 nm minimum investigated) up to the NIR (1000 nm maximum investigated). These spin pairs are universally observed in all samples investigated, and can be coherently driven regardless of wavelength. Finally, we show that $V_{B}^{-}$ defects created by electron irradiation co‐exist with the spin pairs at all wavelengths, and their respective spin states can be independently addressed via appropriate wavelength selection. Our findings establish hBN as a uniquely versatile platform for quantum technologies based on optically addressable spins.

## 2 Results and Discussion

**Figure 1:**
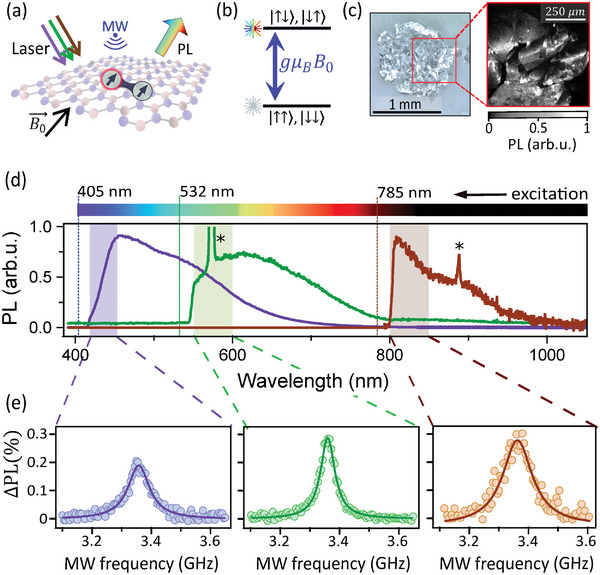
Violet to near‐infrared optical addressing of spin pairs in hBN. a) Schematic representation of a spin pair in hBN excited by different lasers from violet to NIR. Microwave (MW) induced magnetic resonances are detected via a change in photoluminescence (PL) intensity. b) Simplified energy level structure of a weakly coupled spin pair under a magnetic field *B*_0_. The eigenstates are grouped into two effective levels, pure triplet states {|↑↑〉, |↓↓〉} on the one hand and singlet‐triplet mixtures of {|↑↓〉, |↓↑〉} on the other, with an energy separation of *hf*_*r*_ = *g*μ_*B*_ *B*_0_. c)  Photograph of a hBN crystal (left) and corresponding confocal PL map (collection band λ_PL_ = 660 − 760 nm) under 635 nm laser illumination (right). d) Ensemble‐averaged PL spectra from a bright domain of the crystal under three different laser wavelengths: 405 nm (purple line), 532 nm (green), 785 nm (brown). The stars (*) indicate the hBN Raman line. e) ODMR spectra of the spin pairs with an external magnetic field of *B*_0_ ≈ 120 mT for each of the three laser excitation wavelengths. The collected PL bands (shown as shaded areas in (d)) are λ_PL_ = 420–450 nm (left graph, with 405 nm laser), λ_PL_ = 550‐600 nm (middle, 532 nm laser), and λ_PL_ = 800–850 nm (right, 785 nm laser).

The principle of the experiment is depicted in **Figure** **1**a. A laser (wavelength λ_L_) excites an optically active point defect (circled in red) in the hBN sample which in response emits photoluminescence (PL) at wavelengths λ_PL_ > λ_L_. In the presence of a second suitable nearby point defect, under optical pumping the system may form a metastable weakly coupled spin pair with a PL intensity that depends on the state of the spin pair,^[^ ^38^ ^]^ see Figure 1b. When a microwave (MW) field is applied at the resonance frequency of the spin pair, namely *f*_*r*_ = *g*μ_*B*_ *B*_0_/*h* where *g* ≈ 2 is the Landé *g*‐factor, μ_*B*_ the Bohr magneton, *h* Planck's constant, and *B*_0_ the applied magnetic field, the spin state gets mixed and the PL increases.

We first investigate an as‐received bulk hBN crystal sourced from HQ Graphene. A confocal PL image, here showing the red PL emission (660–760 nm), reveals regions with relatively uniform PL extending over tens of microns, separated by darker regions (Figure 1c). These domains are attributed to different levels of carbon incorporation during the growth.^[^ ^40^ ^]^ Throughout the rest of the paper, the PL is collected from a wide (≈50 µm) laser spot in order to average over a large ensemble of emitters. Moreover, we employ three different excitation lasers with λ_L_ = 405 nm (violet), 532 nm (green), and 785 nm (NIR), with the resulting PL spectra shown in Figure 1d. For each laser, the PL is primarily concentrated within 200 nm of the laser wavelength, with a tail extending all the way up to the upper limit of our detector (≈1000 nm). Given most single emitters in hBN have been shown to have sharp but highly variable zero‐phonon lines (ZPLs),^[^ ^15^, ^16^, ^20^, ^21^ ^]^ we interpret these PL spectra as being indicative of the wide distribution of ZPLs contained in the ensemble, convolved with their respective phonon side bands (PSBs).^[^ ^7^ ^]^ The subtle structures in the spectra suggest the ZPLs are not fully evenly distributed, consistent with the analysis by Pelliciari et al.^[^ ^21^ ^]^

To test whether these emitters are associated with an addressable spin pair, we perform continuous‐wave (CW) optically detected magnetic resonance (ODMR) measurements using the PL from different wavelength bands. In Figure 1e, we show ODMR spectra for PL bands close to each laser line, ensuring that ZPLs account for a large fraction of the collected PL: λ_PL_ = 420–450 nm with 405 nm laser, λ_PL_ = 550‐600 nm with 532 nm laser, and λ_PL_ = 800–850 nm with 785 nm laser. In all three cases, a resonance is observed at the expected frequency *f*_*r*_ = *g*μ_*B*_ *B*_0_/*h* ≈ 3.4 GHz (*B*_0_≈120 mT). This result establishes the existence of optically addressable spin pairs with ZPLs from the violet‐blue (420–450 nm) to the NIR (800–850 nm). Moreover, the similar contrast of 0.2–0.3% for the different bands is suggestive of a universal underlying mechanism independent of emission wavelength, supporting the two‐defect model of Robertson et al.^[^ ^38^ ^]^ The wide range of ZPLs likely correspond to several distinct types of emitters (with different atomic structures), although a perturbation of a single type of emitter (e.g., due to strain or nearby charges) may also contribute to the variety.^[^ ^41^ ^]^.

**Figure 2:**
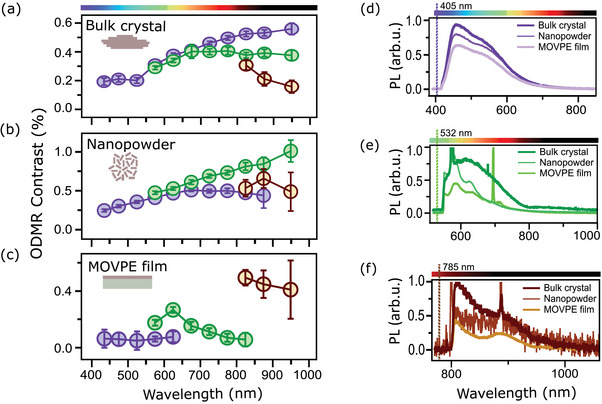
Ubiquity of spin pairs across readout wavelengths and hBN samples. (a–c)  CW ODMR contrast as a function of PL emission wavelength for three different hBN samples (sketched in inset): a) a bulk crystal, b) a nanopowder, and c) a MOVPE‐grown film. For each sample, data from three different excitation lasers are shown: 405 nm (purple markers), 532 nm (green), and 785 nm (brown). Each data point corresponds to a 50‐nm‐wide PL emission band (i.e., 450–500 nm, 500–550 nm, etc.) except for the left‐most point (420–450 nm band) and right‐most point (900‐1000 nm band). Error bars represent the standard error from fitting the ODMR spectrum. (d–f)  PL emission spectra comparing the three hBN samples for each laser wavelength: d) 405 nm, e) 532 nm, and f) 785 nm. The intensity of the spectra was scaled to facilitate comparison between samples while keeping the ordering consistent for ease of readability (from bulk crystal at the top to MOVPE at the bottom).

The ODMR contrast observed from the same bulk hBN crystal is plotted as a function of emission wavelength (within the respective PL spectrum) in **Figure** **2**a for the three lasers. A clear ODMR contrast is measured for all collected wavelengths between the laser line and the upper limit of our detector (≈1000 nm), including with the 405 nm laser. This does not imply, however, that there are ZPLs at all wavelengths, as we are not able to distinguish between ZPL and PSB. For instance, the PL in the 900–1000 nm band could be due to the PSB of emitters with a ZPL close to the 785 nm laser line. Nevertheless, this measurement demonstrates that ODMR can be obtained from PL emission continuously spanning from 420 nm to 1000 nm. Note that the ODMR contrast tends to increase with emission wavelength, for instance going from 0.2% in the blue region to 0.6% in the NIR with the 405 nm laser, which may indicate a higher fraction of ODMR‐inactive emitters in the PL closest to the laser line.

Importantly, we found these results to be reproducible in a wide variety of hBN samples, including bulk crystals sourced from various academic groups, commercial nano/micropowders, and films grown through vapour phase (see SI). In Figure 2b, we show the example of a hBN nanopowder sourced from Graphene Supermarket. Just like the bulk crystal, ODMR contrast is observed for all emission wavelengths from 420 to 1000 nm. Here again the contrast tends to increase with emission wavelength, reaching a maximum of 1.0% at 900–1000 nm under 532 nm excitation. As another example, Figure 2c shows the case of a hBN film grown by metal‐organic vapour‐phase epitaxy (MOVPE).^[^ ^42^, ^43^ ^]^ Again, ODMR is observed for all emission wavelengths from 420 nm to 1000 nm across the three lasers combined, though no ODMR was detected beyond 650 nm with the 405 nm laser and beyond 850 nm with the 532 nm laser. The contrast is generally lower compared to the other samples, and highest with the 785 nm laser, which could indicate a comparatively higher fraction of ODMR‐inactive emitters at lower wavelengths in this sample. The PL spectra from the above three samples are remarkably similar overall, peaking at about 450 nm with the 405 nm laser (Figure 2d), 600 nm with the 532 nm laser (Figure 2e), and 820 nm with the 785 nm laser (Figure 2f). Regular revivals of these peaks are consistently observed which are attributed to PSBs^[^ ^6^, ^7^, ^43^ ^]^ (with the exception of the bulk crystal under 532 nm laser which exhibits a less structured spectrum compared to the other samples, suggesting a different underlying distribution of emitters). From this comparison of very different types of samples, we conclude that hBN universally contains emitters spanning from violet to the NIR, and that regardless of wavelength a fraction of these emitters is associated with an addressable spin pair. This universality lends further support to the model of Robertson et al.,^[^ ^38^ ^]^ which relies on the presence of a suitable nearby defect to explain ODMR rather than on properties of the primary optically active defect itself.

**Figure 3:**
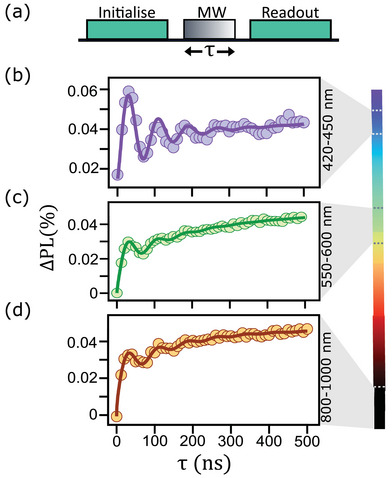
Coherent driving of spin pairs with violet to near‐infrared readout. a) Pulse sequence consisting of a MW pulse of variable duration τ between two 1‐µs laser pulses to initialize and read out the spin state. b–d) Rabi curves acquired for a nanopowder hBN sample by collecting the PL at b) 420‐450 nm under 405 nm laser excitation, c) 550–600 nm under 532 nm laser excitation, and d) 800–1000 nm under 532 nm laser excitation.

Next, we test the ability to coherently drive the state of the spin pairs and optically read them out using the different readout wavelengths. For this, we apply a Rabi sequence where a laser pulse initialises the spin state, followed by a resonant MW pulse of variable duration and a second laser pulse to read out the spin state via the PL (**Figure** **3**a). Example Rabi curves obtained are shown in Figure 3b–d using PL emitted at 420–450 nm, 550–600 nm, and 800–1000 nm, respectively (for these experiments we use the same hBN nanopowder as in Figure 2b). In all cases, clear oscillations are resolved confirming that the spin pairs are sufficiently coherent and long‐lived to sustain Rabi oscillations regardless of the emission wavelength. The systematic rise in the Rabi curves is due to the metastable nature of the spin pair, which decays to the optically active ground state during the MW pulse.^[^ ^38^ ^]^ The more symmetric shape of the Rabi oscillations from the violet‐blue PL may be an indication that the spin pairs have a longer lifetime when associated with these emitters compared to longer‐wavelength emitters,^[^ ^38^ ^]^ motivating future work to study the wavelength‐dependent photodynamics. These results indicate that the spin pairs across all wavelengths are amenable to the application of advanced quantum control protocols,^[^ ^29^, ^30^ ^]^ an important pre‐requisite for many applications.

**Figure 4:**
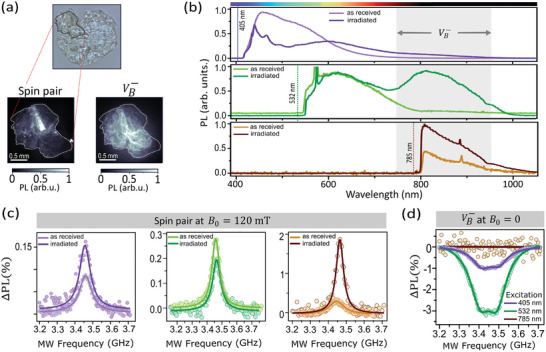
Readout of spin pairs coexisting with $V_{B}^{-}$ defects. a) Photograph (top) and widefield PL images (bottom) of an electron‐irradiated hBN crystal under green LED illumination, filtering for the spin pairs (bottom left, PL emission band λ_PL_ = 600‐700 nm), and the $V_{B}^{-}$ defects (bottom‐right, λ_PL_ = 850‐1000 nm). b) PL emission spectra of the irradiated crystal from (a) compared to an as‐received crystal, for three different laser excitation wavelengths: 405, 532, and 785 nm (top to bottom). c) ODMR spectra of the spin pairs under *B*_0_ ≈ 120 mT, comparing before and after irradiation, for each of the three excitation wavelengths. The collected PL bands are λ_PL_ = 420–450 nm (left graph, with 405 nm laser), λ_PL_ = 550‐600 nm (middle, 532 nm laser), and λ_PL_ = 800–850 nm (right, 785 nm laser). The small frequency shifts between as‐received and irradiated samples are attributed to slight changes in the applied magnetic field between measurements. d) ODMR spectra of the $V_{B}^{-}$ defects in the irradiated crystal under *B*_0_ = 0, for the three excitation wavelengths. The PL collection band is kept the same in all cases (λ_PL_ = 800–1000 nm).

Finally, we investigate the optical readout of spin pairs at various wavelengths in hBN samples where $V_{B}^{-}$ defects are co‐present. This is motivated by the possibility of having multiple spin species independently addressable through different lasers, which would be a useful capability for quantum technologies.^[^ ^36^ ^]^ To create $V_{B}^{-}$, a bulk hBN crystal was irradiated with 2 MeV electrons, which is expected to produce a relatively uniform density of $V_{B}^{-}$ throughout the crystal.^[^ ^44^ ^]^ PL images of the irradiated crystal under green excitation reveal indeed a relatively uniform PL in the NIR attributed mainly to $V_{B}^{-}$, whereas the visible PL from native emitters is more patchy (**Figure** **4**a). In Figure 4b, we compare PL spectra before and after irradiation, for the same three lasers as previously. A broad emission peak centred around 820 nm and characteristic of $V_{B}^{-}$ ^[^ ^22^ ^]^ clearly appears after irradiation when excited with the 532 nm laser (middle graph in Figure 4b). The presence of $V_{B}^{-}$ emission is much less obvious under the 405 nm laser (top graph) which does not excite $V_{B}^{-}$ as efficiently,^[^ ^45^ ^]^ while the 785 nm laser (bottom graph) is close to the ZPL of $V_{B}^{-}$ ^[^ ^46^ ^]^ and does not appear to excite it at all as expected from theory.^[^ ^47^ ^]^ Meanwhile, the native emission is modulated by the irradiation especially in the 450–600 nm emission band but remains present at all wavelengths, consistent with previous work.^[^ ^48^ ^]^.

To verify that the spin pairs can still be addressed after irradiation, we perform ODMR measurements under a magnetic field *B*_0_ ≈ 120 mT, shown in Figure 4c for the three different lasers. The ODMR resonance of the spin pairs is essentially unaffected by the irradiation except for a change in contrast, increasing by about 50% when collecting PL at 420–450 nm under 405 nm laser (left graph in Figure 4c) and decreasing slightly in the 550–600 nm PL under 532 nm laser (middle graph). Interestingly, when collecting the NIR PL (800–850 nm) under 785 nm laser (right graph), the ODMR contrast increases from 0.3% to nearly 2% upon irradiation, which could indicate conversion of ODMR‐inactive emitters (with no suitable nearby defect^[^ ^38^ ^]^) into ODMR‐active ones. To probe the spin resonance of $V_{B}^{-}$, we perform ODMR under zero magnetic field and collect the PL in the 800–1000 nm band. The resulting ODMR spectra from the irradiated crystal are shown in Figure 4d for the three lasers. As expected, the best ODMR contrast (‐3%) is achieved under 532 nm excitation where the PL in the collected band is primarily due to $V_{B}^{-}$ emission.^[^ ^45^ ^]^ The contrast is reduced to –1% with the 405 nm laser as it excites $V_{B}^{-}$ less efficiently. Importantly, there is no evidence of $V_{B}^{-}$ resonance in the ODMR spectrum under 785 nm excitation, confirming this excitation wavelength does not excite $V_{B}^{-}$. These findings suggest a way to decouple the optical addressing of the two spin species, with 785 nm exciting the NIR spin pairs (with maximal ODMR contrast) but not $V_{B}^{-}$, while 532 nm is optimal for $V_{B}^{-}$ readout.

## 3 Conclusion

By performing PL and ODMR spectroscopy on a variety of hBN samples under various laser excitations, we show that hBN universally contains native ODMR‐active optical emitters with emission wavelengths continuously spanning the 420–1000 nm range. The different laser lines employed allow us to determine that, as a minimum, these emitters must have ZPLs in the violet‐blue (405–450 nm), green‐yellow (532–600 nm), and NIR (785–850 nm) regions, which most likely correspond to different types of defects. The ODMR contrast is relatively consistent across emission wavelengths and between samples, supporting the interpretation that ODMR originates from a universal mechanism as we proposed previously,^[^ ^38^ ^]^ involving a metastable weakly coupled spin pair that forms upon charge transfer between the optical emitter (defect A) and a second defect located ≳ 1 nm away (defect B).

Interestingly for future studies and applications of their spin physics, we showed that the spin pairs are sufficiently coherent and long‐lived to sustain Rabi oscillations regardless of the emission wavelength from violet to NIR. Finally, we showed that electron irradiation creates $V_{B}^{-}$ defects without significantly affecting the native ODMR‐active emitters regardless of their emission wavelength (and even improving the NIR‐emitting spin pairs), and identified 785 nm as a convenient laser wavelength to selectively control the NIR spin pairs without driving the $V_{B}^{-}$ spins.

We now take a brief detour to discuss possible microscopic structures for the defects involved. According to the model of Robertson et al.^[^ ^38^ ^]^ defects A and B both must have *S* = 0 in the ground state with defect A additionally optically active, whereas after inter‐defect charge transfer they should each become *S* = 1/2 forming the weakly coupled spin pair. In the as‐received (unprocessed) bulk crystals studied, e.g., in Figure 1, carbon is expected to be the dominant impurity and vacancies negligible.^[^ ^40^ ^]^ It is therefore reasonable to assume the defects are made of carbon, especially substitutional carbon defects (e.g., monomers, dimers, trimers) which are stable and predicted to form under most growth conditions.^[^ ^49^ ^]^ For instance, defect B could be as simple as a carbon monomer ($C_{B}^{+}$ or $C_{N}^{-}$ in the ground state, *S* = 0), while defect A could be one of the many more complex carbon defects previously proposed to explain emission at visible wavelengths, e.g. carbon clusters or double non‐adjacent monomers C_B_‐C_N_ or even possibly C_B_‐C_B_ or C_N_‐C_N_ (with a separation of a few lattice sites), many of them have a *S* = 0 ground state as required by the model.^[^ ^6^, ^7^, ^50^ ^]^ Further work will be required to fully analyze theoretically these defect pair systems and identify which specific defects (possibly multiple ones for defect A to cover different wavelengths) are responsible for our experimental results.

Regardless of the microscopic origin, our results establish the ubiquity of the optically addressable spin pair system in hBN across a uniquely broad range of readout wavelengths. This makes hBN a particularly versatile platform for spin‐based quantum technologies, as the desired wavelength can be chosen to suit each application. For instance, violet emitters may find use in ODMR‐based widefield magnetic imaging^[^ ^51^, ^52^ ^]^ where the shorter wavelength enables an improved spatial resolution compared to existing systems such the nitrogen‐vacancy center in diamond (PL emission centered at 700 nm) or the $V_{B}^{-}$ defect in hBN (820 nm). On the other end of the spectrum, NIR wavelengths are preferable for sensing and imaging of biological samples. Future work will seek to extend the range of wavelengths further in the NIR toward the telecom bands for quantum communication applications, and toward the UV for high‐resolution imaging. The co‐presence of a different spin species ($V_{B}^{-}$) that can be independently addressed may find utility in multi‐modal or multiplexed sensing, or to investigate many‐body spin physics. To advance these various applications, it will be important to progress our understanding of the electronic structure of the spin pair system, and of the atomic structure of the defects involved. This would greatly facilitate the engineering of these systems for applications, and realize the on‐demand creation of single ODMR‐active emitters.

## 4 Experimental Section

### Experimental Setup

The ensemble measurements reported in this work were conducted using a custom‐built room temperature wide‐field fluorescence microscope. Three different laser sources were used, with CW emission at 405 nm (Hubner Photonics Cobolt 06‐01), at 532 nm (Laser Quantum Opus), and at 785 nm (Thorlabs FPL785S‐250), with a laser power at the sample of about 70 mW, 200 mW, and 25 mW, respectively. For pulsed measurements, the CW laser emission was gated either directly or via an acousto‐optic modulator. The gated laser beam was focused through a widefield lens to the back aperture of an objective lens (Nikon S Plan Fluor ELWD 20x, NA = 0.45). Photoluminescence (PL) was separated from excitation light by a dichroic beam splitter optimised for each laser wavelength, filtered using longpass and shortpass filters, and then directed to either a spectrometer (Ocean Insight Maya2000‐Pro) for PL spectroscopy or a sCMOS camera (Andor Zyla 5.5‐W USB3) for spin measurements, where the counts from the entire illuminated region were added together.

The microwave (MW) signal was generated by a Windfreak SynthNV Pro signal generator, gated through a Texas Instruments TRF37T05EVM IQ modulator, and amplified using a Mini‐Circuits HPA‐50W‐63+ amplifier. The amplified signal was fed into a printed circuit board (PCB) with a coplanar waveguide and terminated by a 50 Ω load. The hBN samples were placed directly onto the PCB. A SpinCore PulseBlasterESR‐PRO 500 MHz pulse pattern generator controlled the gating of both the excitation laser and MW, as well as triggered the camera. The static magnetic field was applied using a permanent magnet.

For acquiring the confocal PL maps of bulk crystals shown in Figure 1c and Figure S1 (Supporting Information), a commercial confocal microscope (Olympus FV1200) with CW lasers at 473 nm (15 mW), 559 nm (15 mW), 635 nm (20 mW) was used.

### Sample Preparation

The details of the various samples (bulk crystals, powders, MOVPE film) used in this work are given in Table S1 (Supporting Information). The bulk crystal samples studied in the main text (thickness ∼100 µm, lateral size ∼1mm) were sourced from HQ Graphene. Some of these crystals were irradiated with 2 MeV electrons at a fluence of 2 × 10^18^ cm^−2^ for Figure 4 of the main text. Additionally, in the Supporting Information we show results from a bulk crystal supplied by NIMS, Japan. The powder used in the main text, purchased from Graphene Supermarket (BN Ultrafine Powder), was suspended in isopropyl alcohol (IPA) at a concentration of 20 mg mL^−1^ and horn sonicated for 30 min. The sediment was drop‐cast onto the PCB, forming a continuous film with a thickness of a few microns. A similar method was employed for other commercially sourced powders compared in Figure S2 (Supporting Information). Additionally, the powder studied in the main text was irradiated with 2 MeV electrons at a fluence of 1 × 10^18^ cm^−2^ for studying the creation of $V_{B}^{-}$ defects, shown in Figure S4 (Supporting Information). The MOVPE film was grown on a 2‐inch sapphire wafer by metal‐organic vapor‐phase epitaxy (MOVPE) using triethylboron (TEB) and ammonia.^[^ ^42^ ^]^ The film, grown with a TEB flow of 30 µmol min^−1^, has a thickness of ∼40nm (determined by atomic force microscopy).

## Conflict of Interest

The authors declare no conflict of interest.

## Supporting information

Supporting Information

## Data Availability

The data supporting the findings of this study are available within the paper and its supporting information files.
