# Supplementary material for: Violet to Near‐Infrared Optical Addressing of Spin Pairs in Hexagonal Boron Nitride
Source: Adv Mater. 2025 Feb 18;37(12):2414846. doi: 10.1002/adma.202414846 (PMC11937995; doi:10.1002/adma.202414846)
Supplement: Supplementary file 1 — Supporting Information [file ADMA-37-2414846-s001.pdf]

ADVANCED MATERIALS

Supporting Information

for *Adv. Mater.*, DOI 10.1002/adma.202414846

Violet to Near-Infrared Optical Addressing of Spin Pairs in Hexagonal Boron Nitride

Priya Singh, Islay O. Robertson, Sam C. Scholten, Alexander J. Healey, Hiroshi Abe, Takeshi Ohshima, Hark Hoe Tan, Mehran Kianinia, Igor Aharonovich, David A. Broadway, Philipp Reineck and Jean-Philippe Tetienne**

Supporting Information

I. SAMPLE DETAILS

The samples studied in this work are listed in Table S1, along with the figures in which they were each used. In the main text, bulk crystal 1 sourced from HQ Graphene was used, and another bulk crystal supplied by NIMS was included for comparison in Fig. S2. The hBN nanopowders were sourced from Graphene Supermarket (BN Ultrafine Powder), with a specified purity of 99.0%. Two batches of powder, nominally identical but purchased at different times, were used: 'Nanopowder 1' was purchased in 2017 (used in the main text), whereas 'Nanopowder 2' was purchased in 2022, included for comparison in Fig. S2. Additionally, two micropowders were investigated, with different purities: Micropowder 1 (5 μm particle size, 98% purity) was sourced from Graphene Supermarket, and Micropowder 2 (3-4 μm particle size, 99.99% purity) was sourced from SkySpring Nanomaterials. The MOVPE film, grown with a TEB flow of 30 μmol/min (details can be found in Ref. [1]), has a thickness of $\sim$ 40nm and was identical to that studied in Ref. [2].

Sample	Irradiation	Figures
Bulk Crystal 1<br>(HQ Graphene)	- none<br>- 2 MeV electrons, dose of 5×10^{18}cm^{-2}	- Fig. 1, 2, 3, 4, S1, S3<br>- Fig. 4
Bulk Crystal 2<br>(NIMS)	none	Fig. S2
Nanopowder 1, 99.0% purity<br>(Graphene Supermarket, 2017)	- none<br>- 2 MeV electrons, dose of 1×10^{18}cm^{-2}	- Fig. 2, S2, S3, S4<br>- Fig. S2, S4
Nanopowder 2, 99% purity<br>(Graphene Supermarket, 2022)	none	Fig. S2
Micropowder 1, 98% purity<br>(Graphene Supermarket)	none	Fig. S2
Micropowder 2, 99.99% purity<br>(Skyspring Nanomaterials)	none	Fig. S2
MOVPE film, $\approx$ 40 nm thick<br>grown on sapphire [2]	none	Fig. 2, S2, S3

TABLE S1. List of hBN samples used in this study.

II. CONFOCAL IMAGING OF A BULK CRYSTAL

To optically characterise the bulk crystals and evaluate the spatial homogeneity of photoluminescence (PL) across different excitation wavelengths, confocal PL imaging was performed. While Fig. 1c showed only one wavelength, here we show different illumination and collection conditions for the same region. A 0.6 $\times$ 0.6 mm region of the crystal was analysed (see bright-field image in Fig. S1a) under three excitation wavelengths: 473 nm (blue), 559 nm (green), and 635 nm (red). The corresponding collected emission bands were 500-600 nm, 590-690 nm, and 660-760 nm, respectively (see Fig. S1b-c), selected using appropriate filters in the commercial microscope system.

The PL images of the as-received bulk crystal reveal relatively uniform emission over tens of microns within the analyzed region, with intensities of the same order of magnitude for all three excitation wavelengths. However, direct comparison of PL intensities is challenging due to laser power variations at the focal spot, which were not measured. Spatial variations in PL were observed, with some areas exhibiting higher emission intensities in the red and blue bands compared to the green. These spatial variations could be attributed to the distribution of emitters and their associated ZPLs, as corroborated by the PL spectra shown in Fig. 1.

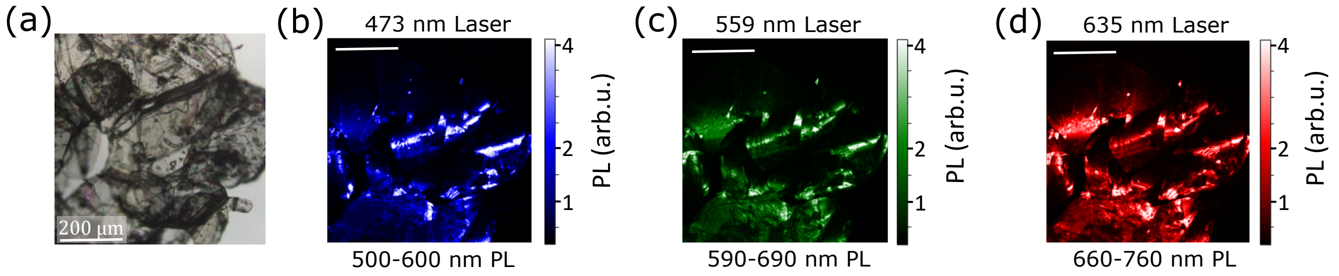

FIG. S1. **Confocal imaging of bulk crystal.** (a) Bright field image of the region of interest. (b) PL map under laser illumination at 473 nm and collection band 500-600 nm of the same region. (c) Laser illumination at 559 nm and collection band 570-670 nm. (d) Laser illumination at 635 nm and collection band 660-760 nm.

III. CHARACTERISATION OF ADDITIONAL SAMPLES

In this section, we further analyse additional samples, specifically powder samples of varying purities sourced from different suppliers, as well as a high quality crystal from NIMS. As shown in Fig. S2a, the PL spectra exhibit a similar shape to the main text samples, with a peak around 600 nm under 532 nm laser excitation. While measurement conditions vary slightly, making direct comparison of PL intensities challenging, the overall spectral similarity confirms the consistency across different samples. It can be further noted that Micropowder 1, which has lower purity compared to the other samples, shows a weaker PL relative to the other powders (as indicated by the noisier spectrum) with an emission peak centered around 650 nm. This shift could be attributed to different emitter distributions caused by impurities in the sample.

Interestingly, when examining the ODMR contrast as a function of emission wavelength under 532 nm excitation, a sign reversal is observed in the Nanopowder 2 sample. For this sample, the relative PL change when the MW is on resonance transitions from approximately (-0.5%) in the 550-600 nm emission range to about +1% in the >900 nm range with a sign flip at about 750 nm (as shown in Fig. S2b,c). Despite these variations, the ODMR effect is reproducible (and typically positive) across all samples, with ODMR detectable from PL emission ranging from 550 nm to 1000 nm (studied under 532 nm and 785 nm only).

Bulk crystal 2 (from NIMS) is also included in Fig. S2a,b, confirming that PL and ODMR can also be observed in a high purity sample. We did not perform a full wavelength-dependent study of ODMR, only collecting the PL over the 550-700 nm range. Bulk crystals sourced from various other academic groups were also studied and gave similar results, not included here.

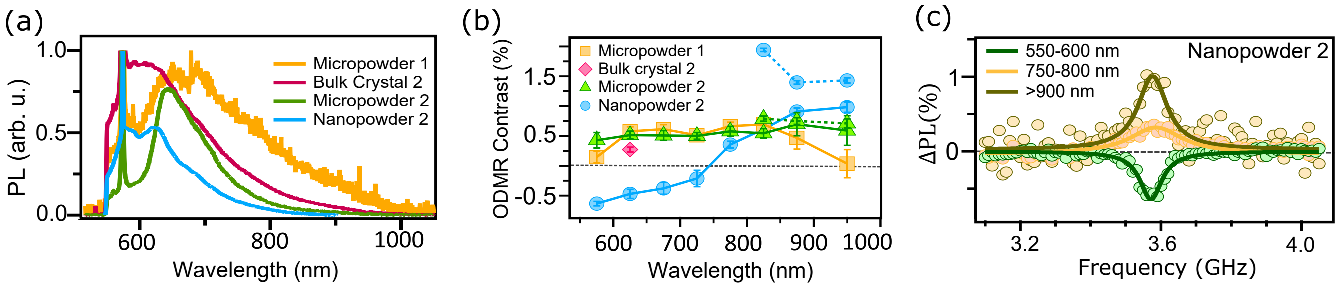

FIG. S2. **PL and ODMR data from additional samples.** (a) PL emission spectra of additional samples under 532 nm laser excitation. (b) CW ODMR contrast as a function of PL emission wavelength for the spin pairs with 532 nm (markers joined by solid lines) and 785 nm (markers joined by dotted lines) illumination. Each data point corresponds to a 50-nm-wide PL emission band (i.e. 550-600 nm, 600-650 nm, etc.) except for the right-most point (900-1000 nm band). For bulk crystal 2, only one ODMR measurement was performed using the 550-700 nm PL. (c) ODMR spectra of the spin pairs in Nanopowder 2 under $B_0 \approx 120$ mT showing positive and negative contrast for different PL emission bands.

IV. ODMR LINEWIDTH VERSUS WAVELENGTH

In Fig. 1e of the main text, the ODMR linewidth appears to depend on the excitation wavelength, being narrowest with 532 nm excitation and broadest with 785 nm excitation. To investigate this effect further, in Fig. S3a we plot the ODMR linewidth as a function of PL emission wavelength for the same bulk crystal as in Fig. 1e. For a given laser, we observe that the linewidth (full width at half maximum, FWHM) does not vary significantly across the PL emission wavelengths, for instance with 405 nm excitation the FWHM remains within 80-100 MHz from the 420-450 nm band to the 900-1000 nm band, compared to about 50 MHz with 532 nm excitation and 130 MHz with 785 nm excitation. Similar plots for the nanopowder and MOVPE film studied in Fig. 2 of the main text are shown in Fig. S3b,c. There is again little variation across emission wavelengths but clear differences between laser, with here the 405 nm laser yielding the broadest linewidth. This difference between lasers even for identical PL emission bands (and MW power kept constant) suggests the laser plays a dominant role in the ODMR linewidth. We leave further investigations of this effect for future work.

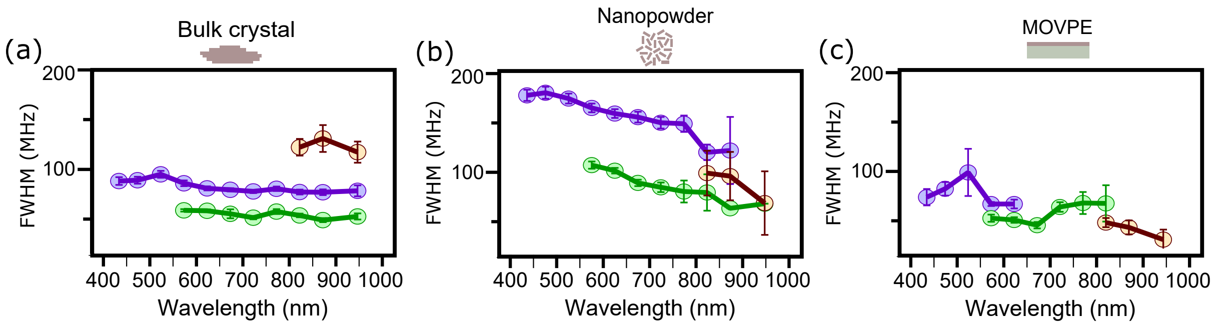

FIG. S3. **ODMR linewidth versus emission wavelength.** (a-c) The full width at half maximum (FWHM) extracted from Lorentzian fits of the CW ODMR spectra with respect to PL emission wavelengths for (a) Bulk crystal 1, (b) Nanopowder 1, (c) MOVPE film. The purple, green and brown lines correspond to the 405 nm, 532 nm and 785 nm laser excitations respectively. All error bars represent the standard errors from the curve fitting. Each data point corresponds to a 50-nm-wide PL emission band (i.e. 450-500 nm, 500-550 nm, etc.) except for the left-most point (420-450 nm band) and right-most point (900-1000 nm band).

V. IRRADIATION OF NANOPOWDER

In Fig. 4 of the main text, we studied the effect of electron irradiation on a bulk crystal. Here we extend our investigation to the case of a nanopowder, and study optical readout of spin pairs where V_B^- defects are present. To generate V_B^- defects, the nanopowder 1 was irradiated with 2 MeV electrons, similar to the bulk crystal. However, due to the increased surface area and higher concentration of defects in the powder, we may expect a different response. First we compare PL spectra of the powder before and after irradiation across the three laser excitations in Fig. S4a-c. Similar to the bulk crystal, a broad emission peak around 820 nm under 532 nm excitation, characteristic of V_B^- defects, is observed but is significantly less prominent which could be due to the lower irradiation dose (by a factor 5). Under the 405 nm (Fig. S4a) and 785 nm (Fig. S4b) the V_B^- emission is also not apparent as expected from the bulk crystal studied in the main text.

ODMR measurements under a magnetic field ($B_0 \approx 120\,\text{mT}$) were performed to verify the presence of the spin pairs post-irradiation. As seen in Fig. S4d, the ODMR resonance of the spin pairs remains largely unaffected by the irradiation except for a change in contrast. Namely, the ODMR contrast increases slightly when collecting PL at 420-450 nm (405 nm laser) and 550-600 nm (532 nm laser), and more significantly it increases from 0.1% to 0.5% after irradiation when examining the NIR PL (800-850 nm) under 785 nm laser excitation (right graph in Fig. S4d). To observe the spin resonance of V_B^-, ODMR was performed under zero magnetic field, with PL collected in the 800-1000 nm band. The resulting ODMR spectra for the irradiated nanopowder 1 sample are shown in Fig. S4e. The 532 nm laser again provides the strongest contrast (-6%) due to efficient excitation of V_B^- defects, higher than that observed in the bulk crystal (-3%). Under 405 nm excitation, the contrast is reduced to -2%, similar to the bulk, due to less efficient excitation of V_B^- [3]. As expected, no V_B^- resonance is detected under 785 nm excitation, confirming that this wavelength does not interact with V_B^- defects even in a nanopowder.

We also attempted to create V_B^- defects in the MOVPE film, however, no signature of V_B^- (whether in PL or ODMR)

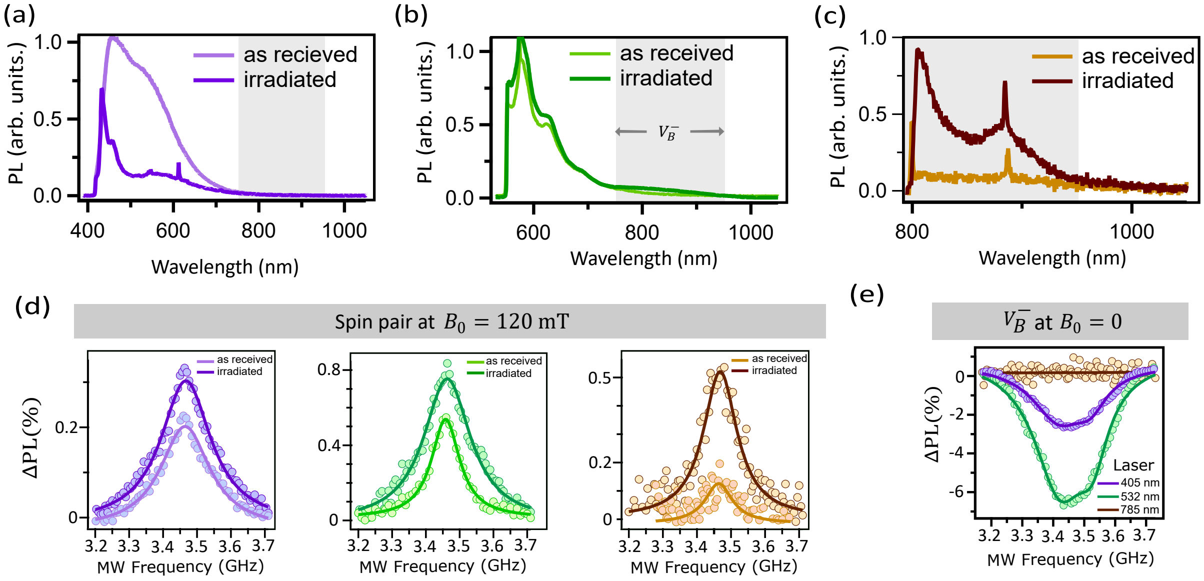

FIG. S4. **Effect of electron irradiation in a nanopowder.** (a-c) PL emission spectra of electron-irradiated nanopowder 1 (see table S1), compared to as-received nanopowder 1, for three different laser excitation wavelengths: (a) 405 nm, (b) 532 nm, and (c) 785 nm. (d) ODMR spectra of the spin pairs under $B_0 \approx 120$ mT, comparing before and after irradiation, for each of the three excitation wavelengths. The collected PL band for each laser is the same as in Fig. 4c. (e) ODMR spectra of the V_B^- defects in the irradiated nanopowder 1 with the same conditions as in Fig. 4d.

was found. This can be explained by the much higher density of luminescent defects in the as-grown MOVPE film (compared to as-received bulk crystals and powders, as estimated by the volume-normalised PL), which overwhelms any contribution from V_B^- defects.

VI. ADDITIONAL RABI CURVES

In Fig. 3 of the main text, we showed Rabi curves for a nanopowder sample (nanopowder 1). Here we show Rabi curves for additional samples, namely: (a) bulk crystal 1, exfoliated into a thin flake to enable fast, uniform MW driving; (b) nanopowder 2; (c) micropowder 2; (d) MOVPE film. The PL band collected is 550-700 nm, under 532 nm laser. The data presented in Fig. S5 reveal Rabi oscillations for all samples except for the MOVPE film, though the latter had weaker MW driving due the substrate standoff. The nanopowder 2 exhibits a reversed contrast as expected for this sample given the negative ODMR contrast observed in Fig. S2b,c.

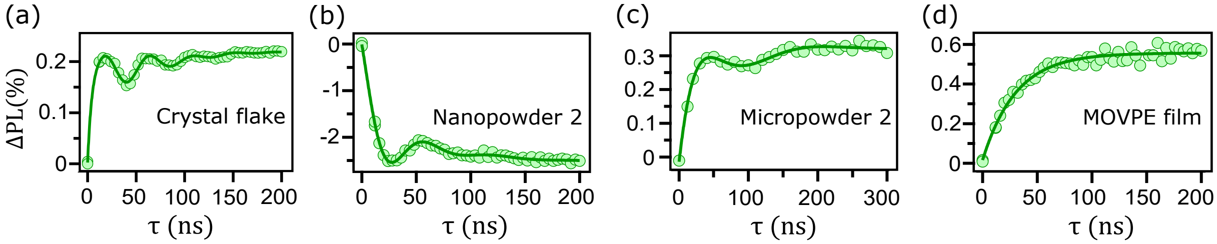

FIG. S5. **Rabi curves in additional samples.** Rabi curves measured at 120 mT under 532 nm laser (550-700 nm PL band) for (a) a flake exfoliated from bulk crystal 1, (b) a film of nanopowder 2; (c) a film of micropowder 2; (d) the MOVPE-grown film. No oscillations are resolved for the MOVPE film, in part due to the weaker MW driving achievable.

[1] D. Chugh, J. Wong-Leung, L. Li, M. Lysevych, H. H. Tan, and C. Jagadish, Flow modulation epitaxy of hexagonal boron nitride, 2D Materials **5**, 045018 (2018).
[2] N. Mendelson, Z.-Q. Xu, T. T. Tran, M. Kianinia, J. Scott, C. Bradac, I. Aharonovich, and M. Toth, Identifying carbon as the source of visible single photon emission from hexagonal boron nitride, Nature Materials **20**, 321 (2021).
[3] M. Kianinia, S. White, J. E. Fröch, C. Bradac, and I. Aharonovich, Generation of spin defects in hexagonal boron nitride, ACS Photonics **7**, 2147 (2020).
